# Central Nervous System Tumors in Xeroderma Pigmentosum: Five Cases and Review of the Literature

**DOI:** 10.1002/mds.70310

**Published:** 2026-05-22

**Authors:** Farrah S. Bakr, Huseyin Arisoy, Alan R. Lehmann, Paola Giunti, Hiva Fassihi

**Affiliations:** ^1^ UK National Xeroderma Pigmentosum Service, St John's Institute of Dermatology, Guy's and St Thomas' NHS Foundation Trust London United Kingdom; ^2^ Ataxia Centre, Department of Clinical and Movement Neurosciences UCL Queen Square Institute of Neurology London United Kingdom; ^3^ Genome Damage and Stability Centre School of Life Sciences, University of Sussex, Falmer Brighton United Kingdom

**Keywords:** DNA‐repair deficiency, glioblastoma, meningioma, primary CNS tumors, xeroderma pigmentosum

## Abstract

**Background:**

Xeroderma pigmentosum (XP) is a rare autosomal recessive DNA‐repair disorder characterized by extreme ultraviolet radiation (UVR) sensitivity, markedly increased cutaneous malignancy risk, and progressive neurological disease in approximately one‐third of patients. Emerging evidence suggests an increased risk of internal malignancies, particularly primary central nervous system (CNS) tumors.

**Objective:**

The aim of this work was to describe primary CNS tumors in patients with XP managed at the UK National XP Service and to review the literature.

**Methods:**

We retrospectively identified five genetically confirmed XP patients with primary CNS tumors followed longitudinally within the multidisciplinary UK National XP Service, including regular neurological evaluation.

**Results:**

Five patients (aged 20–79 years) developed primary CNS tumors: three XP‐C and two XP‐A. Tumors included gliomas, meningiomas, and subependymoma. CNS tumors occurred predominantly in younger patients with XP‐C, whereas late‐onset, slow‐growing meningiomas were observed in milder XP‐A phenotypes. One patient with XP‐C died of glioblastoma.

**Conclusions:**

XP is associated with primary CNS tumor risk, particularly in XP‐C and late‐onset XP‐A, supporting regular neurological assessment and targeted neuroimaging. © 2026 The Author(s). *Movement Disorders* published by Wiley Periodicals LLC on behalf of International Parkinson and Movement Disorder Society.

Xeroderma pigmentosum (XP) is a rare autosomal recessive disorder caused by defective nucleotide excision repair (NER) or translesion synthesis, leading to impaired repair of ultraviolet radiation (UVR)‐induced DNA damage. Nine complementation groups have been described (XP‐A to G, XP‐J, and variant).^1^, ^2^ XP is characterized by marked cutaneous photosensitivity and a substantially increased risk of skin cancer.^3^ In addition to cutaneous malignancy, patients may develop ocular surface disease, lip and oral changes, and internal malignancies.^1^, ^4^ Approximately one‐third of patients develop progressive neurological features, including ataxia, hearing loss, cognitive impairment, and peripheral neuropathy, with severity varying by complementation group and mutation.^5^ Severe early‐onset neurodegeneration is typical of XP‐A, XP‐D, and XP‐G, whereas milder or late‐onset phenotypes occur in other complementations.^1^ Increased prevalence has been reported in consanguineous and isolated populations.^1^ Defective NER also impairs repair of oxidative and chemically induced DNA damage (cyclopurines, chemical carcinogens), which may contribute to the increased risk of internal malignancies, particularly of primary central nervous system (CNS) and hematological tumors.^6^

We report five cases of primary CNS tumors in patients with XP managed within the UK National XP Service, a multidisciplinary center providing coordinated multispecialist care to 130 individuals (Table 1), alongside a review of the literature.

**TABLE 1:** Patients with XP presenting with CNS tumors in this study

Case no.	XP ID no.	Complementation group	Age at XP diagnosis (yr)	Age at diagnosis of brain tumor (yr)	Ethnicity	Allele 1	Protein change	Allele 2^a^	Protein change	Tumor	Age at last observation (yr)	Clinical status at last observation
1	XP28BR	C	3	20	Bangladeshi	c.2176_2192del17	p.Glu726fs	–	–	Low‐grade glioma left posterior temporal lobe	32	Alive; tumor stable on serial MRI
2	XP1SH	C	2	27	White British	c.445_446delGA	p.Glu149fs	c.2336delT	p.Leu779fs	Right intraventricular subependymoma	38	Alive; tumor under surveillance (slow progression)
3	XP21BR	C	6	38	Yemeni	c.658C>T	p.Arg220X	–	–	Grade 4 right parietooccipital glioblastoma	39 (deceased)	Deceased at age 39; aggressive progression of tumor
4	XP134BR	A	57	52	Greek Cypriot	c.389G>A	p.Arg130Lys; abnormal splicing^b^	–	–	Right frontal meningioma	70	Alive; ataxia, cognitive decline, progressive meningioma under monitoring
5	XP1CB	A	?50	79	Indian	c.555+8A>G	Abnormal splicing^c^	–	–	Left cerebellum meningioma	84 (deceased)	Deceased at age 84; ataxia, Parkinson's disease (meningioma stable)

^a^ Indicates homozygous, same mutation as in allele 1.

^b^ Mutation in last base of exon results in abnormal splicing but a small amount of normal splicing.

^c^ Splice mutation results in 5% of normal level of XPA protein^1^.

Abbreviations: XP, xeroderma pigmentosum; CNS, central nervous system; MRI, magnetic resonance imaging.

## Case Reports

### Case 1

A 31‐year‐old Bangladeshi man (XP28BR) with XP‐C, diagnosed at age 3 years after early exposed‐site lentigines, developed multiple basal cell carcinomas from age 8.^1^ Surveillance magnetic resonance imaging (MRI) at age 20 identified a cortical lesion in the left posterior temporal lobe, radiologically consistent with a low‐grade glioma. Biopsy was not performed. The lesion has remained stable on twice‐yearly MRI. Neurological examination showed a stable dystonic upper limb tremor. At age 27, he developed an ocular squamous cell carcinoma treated with excision, cryotherapy, and interferon.

### Case 2

A 36‐year‐old white British man (XP1SH) with XP‐C, diagnosed at age 2 years, developed multiple non‐melanoma skin cancers (NMSCs) from childhood.^1^, ^7^ Baseline brain MRI at age 24 demonstrated a 10‐mm nonenhancing T2‐hyperintense mass arising from the right lateral ventricle, consistent with subependymoma. Diagnosis was radiological, with longitudinal imaging confirming stability, although slight enlargement to 13 mm with peripheral enhancement was noted 5 years later. Biopsy was not performed. Neurological examination showed right‐sided positional and intention tremor with symmetrical reflexes. At age 29, he developed metastatic cutaneous angiosarcoma with lung and nodal involvement. Tumor sequencing demonstrated a high UVR‐driven mutational burden, and he continues to take pembrolizumab because of PD‐L1 expression.

### Case 3

A 39‐year‐old Yemeni woman (XP21BR) with XP‐C, diagnosed at age 6 years after presenting with lentigines, had a history of multiple NMSCs and a nasal melanoma treated with surgery and radiotherapy.^1^ At age 38, she presented with headache, fatigue, and left arm weakness. MRI showed a right parieto‐occipital lesion, and biopsy confirmed grade 4 glioblastoma multiforme. She received temozolomide, radiotherapy, and dexamethasone. Despite treatment, rapid tumor progression occurred with a contiguous new lesion, and she died 10 months after diagnosis before this lesion could be biopsied.

### Case 4

A 69‐year‐old Greek Cypriot man (XP134BR) with XP‐A was diagnosed at age 57 years after progressive personality change and cerebellar ataxia beginning in his mid‐40s.^8^ He reported lentigines since childhood and developed multiple melanomas from age 59. MRI demonstrated cerebellar and brainstem atrophy and a right frontal meningioma extending into the sphenoid, optic nerve, and chiasm. The meningioma has shown gradual enlargement with increasing edema, particularly over the past 4 years. Biopsy was not done. He developed chorea, cognitive impairment, and hearing loss requiring aids, alongside progressive ataxia.

### Case 5

A man of Indian origin (XP1CB) with genetically confirmed XP‐A presented to our service at age 76 years.^1^, ^9^ He had multiple NMSCs and *in situ* melanomas from his 60s and progressive hearing impairment. Examination at age 77 demonstrated cerebellar signs with mild bilateral parkinsonism. MRI at age 78 showed a stable extra‐axial left cerebellar meningioma (23 × 13 mm), global cerebellar atrophy, and iron deposition in the substantia nigra. Biopsy was not done. He died at age 84 of Parkinson's disease–related complications.

## Discussion

In addition to an increased incidence of skin cancers, patients with XP have a higher frequency of internal malignancies compared with the general population, most commonly tumors of the CNS and hematological malignancy.^6^ From 1947 to date, 14 cases of CNS tumors occurring in patients with XP have been reported in the literature (Table 2).^6^ All patients in this series were reviewed regularly within a multidisciplinary XP service, including neurological assessment, which facilitated early detection and careful longitudinal evaluation of CNS lesions. Similar mild or slow‐growing CNS tumors may therefore be underrecognized in patients with XP not followed within structured multidisciplinary service. In this report, we report a further five cases: three patients with XP‐C and two patients with XP‐A. Patients with XP‐C were younger with a median age of 23.5 years versus 65.5 years in patients with XP‐A.

**TABLE 2:** Patients with XP presenting with CNS tumors in the literature

Reference	XP ID no.	Complementation group	Age at diagnosis of tumor (yr)	Tumor
Thomas (1947)^14^	–	–	33	Brain sarcoma
Robbins (1974)^15^	XP14BE XP15BE	C C	N/A 16	Schwannoma Glioblastoma
Fuji (1979)^16^	–	–	9	Astrocytoma
Giannelli (1981)^17^	XP106LO	C	14	Medulloblastoma
Kraemer (1984)^18^	–	–	16	Brain sarcoma
Satoh (1988)^19^	–	A	8	Glioblastoma
Nakamura (1991)^20^	XP97TO	D	43	Schwannoma
Digiovanna (1998)^21^	XP23BE	C	22	Spinal cord astrocytoma
Giglia (1998)^11^	XP233VI	C	7	Thalamic glioma
El Ouazzani Chahdi (2010)^22^	–	–	35	Schwannoma
Lai (2013)^23^	XP24BE	C	29	Glioblastoma
Plante (2021)^24^	–	–	18	Diffuse midline glioma
Nikolaev (2022)^6^	XPAdSaVI	C	14	Astrocytoma

Abbreviations: XP, xeroderma pigmentosum; CNS, central nervous system; N/A, not available.

Of the 21 patients with XP‐A in our cohort, cases 4 and 5 are the only individuals older than 50 years, both demonstrating late‐onset neurological features beginning after age 45.^5^ Their milder phenotypes are attributable to hypomorphic variants: a missense mutation with abnormal splicing (c.389G>A, p.Arg130Lys) in case 4^8^ and a cryptic splice‐site variant (c.555+8A>G) in case 5.^1^ Both developed slow‐growing meningiomas after age 50.^1^ In both, neurodegenerative features preceded tumor detection, suggesting that ataxia, cognitive impairment, and parkinsonism were primarily related to underlying XP‐A–associated neuronal pathology rather than mass effect, although behavioral change in case 4 may have been influenced by frontal tumor location. These findings underscore the need to distinguish intrinsic XP‐related neurodegeneration from tumor‐related neurological deficits when evaluating new or progressive symptoms.

The occurrence of meningiomas exclusively in these late‐onset XP‐A cases suggests that residual NER activity and extended survival permit development of age‐related CNS neoplasms. Classical XP‐A patients with severe early‐onset neurodegeneration rarely survive into later adulthood and are infrequently reported to develop CNS tumors. The apparent enrichment of meningiomas in mild XP‐A may reflect the combined effects of altered DNA‐repair capacity and increased lifespan rather than mutation‐specific susceptibility. Notably, meningiomas were observed only in these two atypically mild cases among 43 XP patients older than 45 years within our service.

A coordinated multicenter registry study would be valuable to better define whether meningioma risk differs across XP complementation groups. Systematic collection of longitudinal clinical and neuroimaging data across international XP cohorts would allow comparison of CNS tumor incidence according to complementation group, mutation type, and neurological phenotype. Integration of genetic, radiological, and clinical outcome data would also help determine whether specific variants confer differential tumor risk.

In our cohort, most of the primary CNS tumors were seen in patients with XP‐C. Three of the 32 patients with XP‐C were affected. This is consistent with the literature where seven of nine patients for whom complementation group data are available had XP‐C (Table 2). The most common primary CNS tumor was a glioma. Patients with XP‐C are also reported to have a higher incidence of hematological malignancies compared with other complementation groups.^10^ Therefore, we postulate that deficient NER mechanisms play a key role in the development of internal tumors in the younger XP‐C cohort.

Notably, the patients with XP‐C in our cohort did not demonstrate classical early‐onset progressive neurodegeneration as described in severe XP‐A or XP‐D phenotypes.^1^, ^5^ Although mild tremor was observed in cases 1 and 2, there was no evidence of significant childhood‐onset hearing loss, intellectual decline, or progressive cerebellar syndrome preceding tumor detection. This contrasts with the two patients with XP‐A, who exhibited adult‐onset neurodegenerative features including ataxia, chorea, cognitive impairment, and parkinsonism. These findings align with established genotype‐phenotype differences, whereby XP‐C is associated with malignancy risk but limited primary neurodegeneration.^1^, ^5^

An apparent paradox exists between neurodegeneration severity and primary CNS tumor risk across XP complementation groups. Patients with severe early‐onset neurodegeneration (classic XP‐A/XP‐D/XP‐G) rarely develop primary CNS neoplasms, whereas XP‐C patients—who typically lack significant early neurodegeneration—account for most CNS tumors described in the literature and in our cohort. This supports a role for endogenous oxidative DNA damage, rather than UVR exposure, in internal tumorigenesis.

The mechanisms underlying increased internal tumor risk in XP remain incompletely defined. Analysis of internal tumors in patients with XP‐C has demonstrated p53 mutations distinct from UVR‐signature mutations seen in cutaneous cancers, including G→T and A→T substitutions rather than CC→TT transitions.^11^ This suggests that endogenous oxidative DNA damage, rather than UVR exposure, contributes to internal tumorigenesis. Impaired repair of oxidatively induced lesions, including 8,5′‐cyclopurine‐2′‐deoxynucleosides and 8‐oxoguanine, may lead to accumulation of DNA damage in internal tissues.^12^

Experimental studies show that XP‐C cells accumulate cyclopurines and demonstrate reduced repair of oxidized bases.^11^ Elevated oxidative lesions and ubiquitin‐proteasome system dysregulation have also been identified in XP neurons.^13^ Together, these findings support a model in which defective repair of endogenous oxidative damage contributes to both neurodegeneration and internal tumor susceptibility.

The heterogeneous clinical course and tumor behavior observed across our cases likely reflect the interplay of several factors: complementation group, residual DNA‐repair capacity, mutation type, age at presentation, and tumor histology. Patients with XP‐C in our cohort developed predominantly gliomatous tumors at younger ages, consistent with more pronounced genomic instability in proliferating tissues. In contrast, the patients with XP‐A with hypomorphic or splicing variants survived into later adulthood and developed slow‐growing extra‐axial meningiomas. These differences suggest that both the degree of residual NER function and cumulative lifespan exposure may influence tumor phenotype and timing. Integrating genetic subtype, neurological severity, and tumor characteristics may improve risk stratification in XP.

A limitation of this study is that histological confirmation was obtained only in case 3. The remaining tumors were diagnosed based on characteristic radiological features and longitudinal imaging stability. Although imaging findings were characteristic, the absence of histopathological confirmation means atypical neoplasms cannot be fully excluded, reflecting cases in which biopsy was not clinically indicated.

In summary, we present five XP cases with primary CNS tumors. Patients with XP also have a high frequency of malignancies, including melanoma, which may metastasize to the CNS.^1^, ^3^, ^6^ Endogenous oxidative DNA damage may contribute to this process.

Given the distribution of tumors, regular neurological evaluation within specialized multidisciplinary services is advisable. Baseline brain MRI in early adulthood for patients with XP‐C, with repeat imaging guided by new or progressive neurological symptoms, may be reasonable. In patients with late‐onset XP‐A, particularly those with evolving neurological features or hypomorphic variants, neuroimaging should be individualized, with early imaging in the presence of focal deficits, accelerated cognitive decline, or atypical progression beyond the expected XP‐related neurodegenerative course.

## Author Roles

1. Research Project: A. Conception, B. Organization, C.Execution; 2. Statistical Analysis: A. Design, B. Execution, C. Review andCritique; 3. Manuscript: A. Writing of the First Draft, B. Review and Critique.

F.S.B.: 1A, 1B, 1C, 2A, 2B, 2C, 3A.

H.A.: 1A, 1B, 1C, 2A, 2B, 2C, 3A.

A.R.L.: 3A, 3B.

P.G.: 1A, 1B, 1C, 2A, 2B, 2C, 3A, 3B.

H.F.: 1A, 1B, 1C, 2A, 2B, 2C, 3A, 3B.

## Financial Disclosures and Conflicts of Interest

P.G. has received personal fees from UCB and Biogen for consultancy services, outside the submitted work. P.G. has participated on the advisory board for Biogen. The authors report no competing interests. Author disclosures are available in the Supporting Information.

## Data Availability

The data that support the findings of this study are available from the corresponding author upon reasonable request.
